# Prognostic Value of Components of Body Composition in Patients Treated with Targeted Therapy for Advanced Renal Cell Carcinoma: A Retrospective Case Series

**DOI:** 10.1371/journal.pone.0118022

**Published:** 2015-02-10

**Authors:** Weijie Gu, Yao Zhu, Hongkai Wang, Hailiang Zhang, Guohai Shi, Xiaohang Liu, Dingwei Ye

**Affiliations:** 1 Department of Urology, Fudan University Shanghai Cancer Center, Shanghai, China; 2 Department of Radiology, Fudan University Shanghai Cancer Center, Shanghai, China; 3 Department of Oncology, Shanghai Medical College, Fudan University, Shanghai, China; University of British Columbia, CANADA

## Abstract

**Background:**

To evaluate the association between various components of body composition and overall survival of patients treated with targeted therapies for advanced renal cell carcinoma.

**Methods:**

This retrospective study included 124 Chinese patients with advanced renal cell carcinoma who had been treated with targeted therapy from 2008 to 2012 at Fudan University Cancer Center. The L3 plane from a computed tomography scan was analyzed. Area and density were recorded as quantitative and quality measures. Univariate and multivariate Cox proportion hazard regression models were constructed to calculate the crude and adjusted hazard ratio (HR) of various components of body composition for overall survival. X-tile software was used to search for optimal cutoffs for male and female patients and the concordance index evaluated incremental changes in prognostication.

**Results:**

After adjusting for age, sex and Heng risk stratification, only visceral adipose tissue index (HR 0.981, p = 0.002) and subcutaneous adipose tissue index (HR 0.987, p = 0.048) were independently associated with overall survival. Visceral adipose tissue remained a significant prognostic factor (HR 0.997, p = 0.005) when the influence of body mass index was included. Using defined cutoffs, patients with low VAT had double the death rate (p = 0.007). Visceral adipose tissue also added significant benefit to Heng risk stratification. Further exploratory analysis revealed that visceral adipose tissue may be an indicator of nutritional status in patients with advanced renal cell carcinoma.

**Conclusion:**

Radiologic measurement of VAT is an independent prognostic factor for Asian patients treated with targeted therapy for advanced renal cell carcinoma.

## Introduction

The incidence of renal cell carcinoma (RCC) has been increasing over the last decade: RCC now accounts for approximately 2–3% of all malignancies worldwide.[1] Although an increasing proportion of patients have small tumors at diagnosis, a quarter still present with locally advanced or metastatic disease. Furthermore, after surgical removal of renal tumors, recurrence occurs in one third of patients.[2] Over the last decade, better understanding of molecular aspects of carcinogenesis has led to the development of targeted therapies for RCC. Despite remarkable improvements in disease control and survival benefit, advanced disease is still incurable with a median survival of 2 years.[3] Therefore, optimizing clinical assessment, tailoring existing therapies, and designing future clinical trials are areas of high-priority research. Because it would be helpful in achieving these goals, identifying prognostic factors is highly desirable. Heng et al. have developed a prognostic scoring system that uses easily obtained clinical and laboratory variables.[4] The model has been successfully validated in a population-based study and is now considered a fundamental step toward personalized targeted therapy.[5]

Progression of cancer generally reflects an imbalance between tumor and host.[6] Although much research has addressed the characteristics of tumors, little attention has been paid to host factors. Anthropometric body measurement provides detailed information about body composition, which indicates nutritional status, capacity to metabolize drugs, and endocrine function.[7] These factors play a crucial role in the causation, prognosis and treatment outcome of cancer. Although several studies have explored the role of body mass index (BMI) in RCC, little other research has investigated body composition in this context.[8]

Ladoire et al. first reported that visceral obesity has significant prognostic value in patients with advanced RCC treated with target therapy.[9] They found that shorter survival correlated with increased visceral obesity in 64 European patients. Interestingly, another European group similarly assessed 116 subjects and came to the opposite conclusion: more than average adipose tissue was associated with longer survival.[10] These two studies used the Memorial Sloan-Kettering Cancer Center criteria in their multivariate analysis. Subsequently, Antoun et al. evaluated the prognostic value of body composition adjusted by Heng risk score [11] and found no statistically significant associations between quantitative measurements and survival. On the other hand, in their cohort they found that skeletal muscle density was an independent prognostic indicator. In light of these contradictory data, and the need for suitable prognostic factors for other races, we evaluated associations between quantitative and quality measures of body composition and overall survival of patients with advanced RCC treated with targeted therapies. Our aim was to determine whether such data provide additional prognostic value.

## Patients and Methods

### Study participants

This retrospective study included 124 Chinese patients with advanced RCC who had been treated with targeted therapy from 2008 to 2012 at Fudan University Cancer Center (Shanghai, China). All subjects had advanced RCC, complete BMI data and underwent pretreatment abdominal computed tomography (CT) scans. The indication for targeted therapies had been the presence of metastatic disease. Baseline clinical and laboratory data previously found to have prognostic value were collected as described previously.[5, 9, 10, 12] Outcome data on overall survival (OS) were obtained from a prospectively maintained database. Progression-free survival was not used as a study endpoint because independent radiological evaluation was not available for some patients. Heng risk score was used to stratify these patients. This originally three-tier risk stratification system was simplified into two tiers because of the small proportion (4.8%) of high-risk patients in this cohort. Intermediate- and high-risk patients were combined to form a single group termed HI-risk.

This study was approved by the review board of Fudan University Shanghai Cancer Center and all patients gave written informed consent.

### Anthropometry and body composition measurements

Weight and height were measured before initiation of treatment using calibrated instruments. BMI was calculated by the following formula: BMI = weight/height^2^. Body surface area (BSA) was calculated by the Mosteller formula: BSA = square root (weight × height/3600).[13]

Visceral (VAT) and subcutaneous (SAT) adipose tissue and skeletal muscle were assessed using digital CT images stored in a picture archiving and communications system. The third lumbar vertebra (L3) section was chosen as the working plane because it has been demonstrated to be linearly correlated to whole-body skeletal muscle and adipose tissue masses.[14]

First, images were analyzed using pre-established Hounsfield unit thresholds (−190 to −30 for fat, −29 to 150 for skeletal muscle), then the regions of interest were manually outlined and VAT, SAT and SM lumbar cross-sectional areas (cm^2^) automatically calculated by ImageJ software.[15, 16] These values were normalized for stature and expressed in units of cm^2^/m^2^, as described in a previous study.[14] Second, the mean radiation attenuation of VAT, SAT, and skeletal muscle was recorded and a similar method used to evaluate the density of adipose tissue and skeletal muscle.[11] All measurements were performed by two trained radiologists who were blinded to patient information, clinical treatments and outcomes. Mean values for each subject were used in the final analysis.

### Statistical analysis

The primary endpoint of this study was OS time, which was defined as the time from targeted agent administration to the date of death or last contact. The follow-up duration was calculated using a reversed Kaplan—Meier method.[17] Continuous variables are reported as medians and interquartile ranges (IQRs) and categorical data as proportions. The Shapiro—Wilk test was used to test whether the data were normally distributed.

To evaluate the prognostic value of investigated variables, hazard ratios (HRs) and 95% confidence intervals (CIs) were calculated using the Cox proportional hazards model. Three steps were followed: first, crude HRs were calculated; then adjusted prognostic values were evaluated by taking into account age, sex, and Heng risk stratification (low-risk vs. HI-risk groups); and finally, whether prognostic values were still independent after inclusion of BMI was determined.

The OS was determined using the Kaplan—Meier method with Rothman 95% CI and compared across groups using the log-rank test. X-tile software was used to further analyze the optimal cutoffs for male and female patients.[18] Once the prognostic factors had been identified, they were added to the Heng model to form a new model. The predictive accuracy of the models was evaluated by the concordance index (C-index). The LR χ² test for nested models was used to assess whether new variables added predictive value to the baseline models.

An exploratory analysis was also performed to test the hypothesis that VAT index is an indicator of nutritional status in advanced RCC patients. This involved further analyzing correlations between VAT index and clinical characteristics and malnutrition inflammation scores (MIS) by two-sample *t*-test and one-way analysis of variance. The MIS was constructed according to previously published reports and data availability.[19, 20]

All analyses were performed using R software. *P*-values <0.05 were considered significant in all tests. All *P*-values are two-sided.

## Results

### Patient characteristics

Detailed patient characteristics are summarized in Table 1. The patients received vascular endothelial growth factor (VEGF)-targeted therapy (sunitinib, *n* = 52; sorafenib, *n* = 26; axitinib, *n* = 14; pazopanib, *n* = 8, famitinib, *n* = 10), and inhibitors of the mammalian target of rapamycin or mTOR (everolimus, *n* = 7). Targeted therapy was administered as first-line treatment in 68 patients (54.8%). Most patients had good performance status (Karnofsky performance status ≥ 80, 90.3%). According to the Heng risk score, 29% of the patients were low risk (no risk factors, 36 patients), 66.1% intermediate risk (one to two risk factors; 82 patients), and 4.8% high risk (three or more risk factors, six patients). According to BMI, eight patients (6.5%) were considered to be malnourished (BMI of <18.5 kg/m^2^). The normality test showed that body composition measurements were normally distributed (data not shown). CT measurements of adiposity and skeletal muscle showed good interoperator reliability with an interclass correlation coefficient of 0.96.

**Table 1:** Baseline patient characteristics (n = 124).

Variables	Value (IQR)
**Age**, yr	58 (51~64)
Gender	
Male	80 (64.5%)
Female	44 (35.5%)
KPS	
≥80	112 (90.3%)
<80	12 (9.7%)
Heng risk score	
Low	36(29%)
Intermediate	82(66.1%)
High	6(4.8%)
**BMI**, Kg/m^2^	22.94 (20.44~25.52)
**BSA**, m^2^	1.73 (1.56~1.87)
**SM index**, cm^2^/m^2^	45.04 (39.92~50.03)
**SM density**, HU	7.04 (-5.03~-19.60)
**VAT index**, cm^2^/m^2^	40.84 (19.39~55.82)
**VAT density**, HU	-56.26 (-63.14~-49.50)
**SAT index**, cm^2^/m^2^	43.85 (28.41~54.41)
**SAT density**, HU	-92.75 (-96.93~-87.08)

Abbreviations: IQR, interquartile range; KPS, Karnofsky performance status; BMI, body mass index; BSA, body surface area; SM, skeletal muscle; VAT, visceral adipose tissue; SAT, subcutaneous adipose tissue; HU, Hounsfield unit.

### Body composition measurements and overall survival

During a median follow-up of 30.8 months (95% CI: 26.4–35.2 months), 67 patients (54.03%) died. The median OS time was 24.7 months (95% CI: 19.7–34.8 months).

According to univariate Cox regression analyses, four body composition variables were significantly associated with OS (Table 2). VAT index (HR: 0.980, 95% CI: 0.969–0.991, *p* = 0.001), SAT index (HR 0.986, 95% CI: 0.973–0.999, *p* = 0.037), BMI (HR: 0.910, 95% CI: 0.838–0.987, *p* = 0.024), and BSA (HR: 0.244, 95% CI: 0.066–0.908, *p* = 0.035) were all positively correlated with survival. When adjusted for age, sex, and Heng risk stratification, VAT index (HR: 0.981; 95% CI, 0.969–0.993, *p* = 0.002) and SAT index (HR: 0.987, 95% CI: 0.974–1.000, *p* = 0.048) were identified as independent prognostic factors for OS. In contrast, BMI (*p* = 0.121) and BSA (*p* = 0.335) failed to show strong associations with patients’ outcome. After further adjusting for BMI, VAT index was identified as the only significant prognostic factor (HR: 0.997, 95% CI: 0.994–1.000, *p* = 0.005).

**Table 2:** Cox regression models analyzing the potential influence of body composition variables on OS times.

Variable	HR of Death					
	Crude		Adjusted by Heng, age, and gender		Adjusted by Heng, age, gender, and BMI*	
	HR (95% CI)	p	HR (95% CI)	p	HR (95% CI)	p
VATI	0.980(0.969–0.991)	0.001	0.981(0.969–0.993)	0.002	0.997(0.994–1.000)	0.005
SATI	0.986(0.973–0.999)	0.037	0.987(0.974–1.000)	0.048	1.000(0.999–1.000)	0.141
SMI	0.998(0.968–1.028)	0.882	0.996(0.960–1.003)	0.846		
BMI	0.910(0.838–0.987)	0.024	0.94(0.861–1.018)	0.121		
BSA	0.244(0.066–0.908)	0.035	0.441(0.083–2.329)	0.335		
VATD	1.013(0.988–1.039)	0.303	1.009(0.982–1.035)	0.528		
SATD	1.000(0.990–1.010)	0.997	1.007(0.997–1.017)	0.171		
SMD	0.994(0.981–1.007)	0.376	1.000(0.986–1.013)	0.950		

Abbreviations: HR, hazard ratio; VATI, visceral adipose tissue index; SATI, subcutaneous adipose tissue index; SMI, skeletal muscle index; BMI, body mass index; BSA, body surface area; VATD, visceral adipose tissue density; SATD, subcutaneous adipose tissue density; SMD, skeletal muscle density

* interaction term with BMI was included

**Fig 1:**
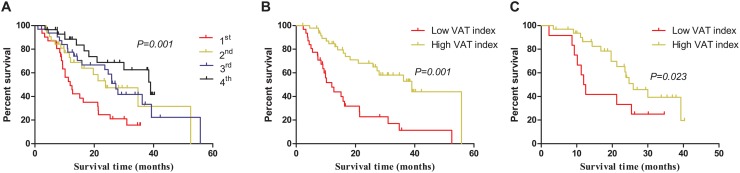
Kaplan—Meier assessment of overall survival according to visceral adipose tissue (VAT) index. A: Kaplan—Meier assessment of overall survival when VAT index was quartered. B: Kaplan-Meier assessment of overall survival in male patients when VAT index was dichotomized by 33.3 cm^2^/m^2^. C: Kaplan-Meier assessment of overall survival in female patients when VAT index was dichotomized by 17.7 cm^2^/m^2^.

To make a preliminary evaluation of the clinical utility of the VAT index, this continuous variable was quartered. According to Kaplan—Meier analysis, the median OS in patients with high (fourth quartile) VAT index was threefold longer than that of patients with low (first quartile) VAT index (38.4 months vs. 11.9 months, *p* = 0.001, log-rank test) (Fig. 1A). Analysis of survival data with X-tile revealed optimal cutoff points of 33.3 cm^2^/m^2^ and 17.7 cm^2^/m^2^, which are identical to those established for VAT index for male and female patients. As shown in Fig. 1B, male patients with low VAT index had a median OS of only 12.3 months, whereas those with high VAT index had a median OS of 39.0 months (*p* < 0.0001, log-rank test). Fig. 1C showed similar results for female patients (25.9 months vs. 12.2 months, *p* = 0.023, log-rank test).

Whether VAT index provides better risk stratification was also explored. In the new model, patients with low VAT index have double the risk of death (Table 3). A significant increase in C-index was observed when VAT index was added to the previous six independent variables of the Heng classification. The likelihood ratio test showed a statistically significant improvement (*p*<0.001). After using a step-wise backward method, the final model included low VAT index, KPS < 80%, hemoglobin < lower limits of normal and calcium > upper limits of normal. The HR of the VAT index was between those of hemoglobin and calcium (S1 Table).

**Table 3:** Multivariate Cox regression models analyzing visceral adipose tissue index, Heng model classification and overall survival.

Parameter	Hazard Ratio	95% CI	*P*-value
**Heng model**			
KPS < 80%	2.194	1.022–4.707	0.044
Time from diagnosis to treatment < 1 year	1.225	0.717–2.090	0.458
Hemoglobin < LLN	2.048	1.234–3.567	0.007
Calcium > ULN	1.546	0.595–4.017	0.371
Neutrophil count > ULN	1.421	0.613–3.299	0.413
Platelet count > ULN	1.721	0.888–3.337	0.108
Body composition			
Low VAT index*	2.087	1.222–3.566	0.007

**Performance**	**Heng model**	**Adding VAT index**	
C-index	0.674	0.699	

Abbreviations: VAT, visceral adipose tissue; KPS, Karnofsky performance status; LLN, lower limits of normal; ULN, upper limits of normal; C-index, Harrell’s concordance index

*VAT index less than 33.3 cm^2^/m^2^ for male or 17.7 cm^2^/m^2^ for female

A further subset analysis was performed on patients (n = 86) who had received sunitinib or sorafenib therapy. The findings were similar: a low VAT index indicated poor prognosis after adjustment by the factors from Heng risk stratification (HR: 1.987, 95% CI: 1.045–3.736, *p* = 0.025).

**Fig 2:**
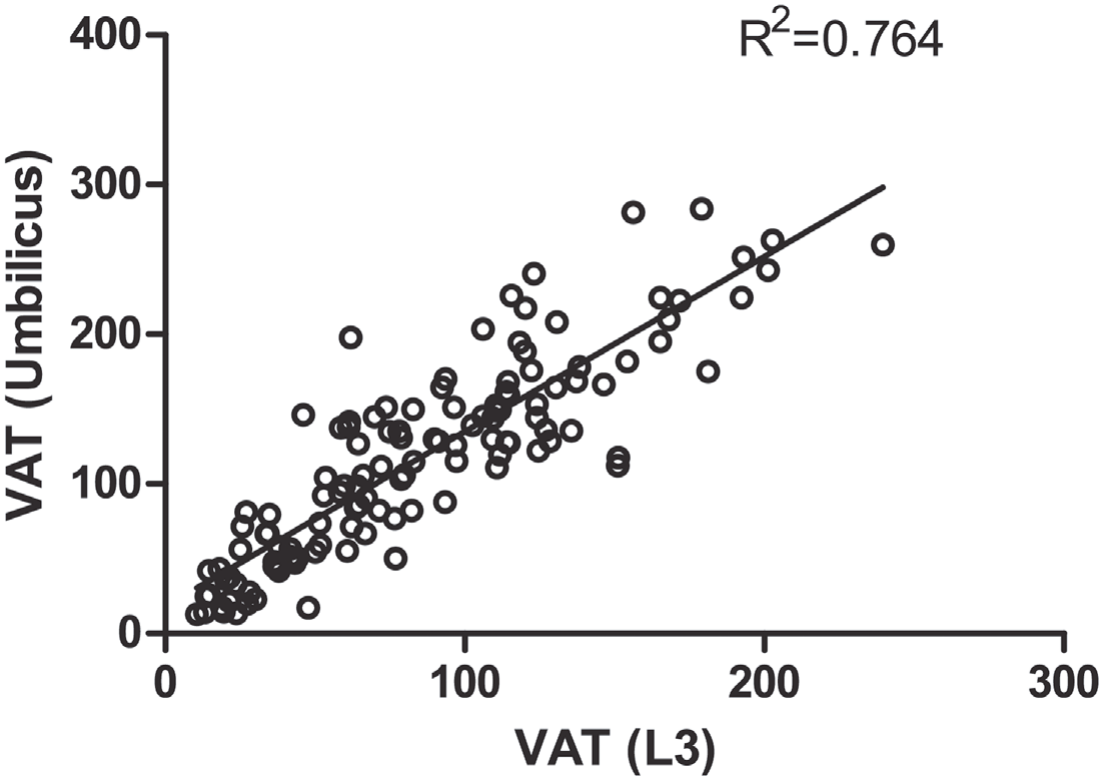
Correlation of visceral adipose tissue area between the level of L3 and umbilicus in the supine position.

The variables of body composition were also measured at the level of the umbilicus (approximately the level of L4–L5) with patients in the supine position; these were fairly well correlated with those for the level of L3 (Fig. 2).

### Exploratory analyses of correlation of VAT index with nutrition-related factors

Patients with higher VAT index tended to be older and male (*p* = 0.033, 0.224, respectively). Prior systemic treatment and nephrectomy did not significantly influence VAT index (*p* = 0.152, 0.961, respectively). VAT index was significantly lower in patients with anemia (*p* = 0.001). Similarly, VAT index was likely to be low in patients with low serum albumin concentrations (41.77 vs. 34.06, *p* = 0.270) and high neutrophil:lymphocyte ratios (36.98 vs. 43.41, *p* = 0.151). These three variables (hemoglobin, albumin, neutrophil:lymphocyte ratio) make up the MIS. Lower MIS was associated with a trend toward decreased VAT index (47.68 for none of these risk factors vs. 36.48 for one of them vs. 32.54 for two or more, *p* = 0.01) (Table 4).

**Table 4:** Correlations between VAT index, clinical characteristics and malnutrition inflammation scores.

	N (%)	VATI mean (S.E)	*p*-value
Clinical characteristics			
Age			0.033
>60	48(38.7%)	46.59(3.61)	
≤60	76(61.3%)	37.21(2.59)	
Gender			0.224
Male	80(64.5%)	42.79(2.81)	
Female	44(35.5%)	37.29(3.19)	
KPS			0.702
≥80	112(90.3%)	41.23(2.27)	
<80	12(9.7%)	38.21(7.39)	
Prior systematic treatment			0.140
No	68(54.8%)	23.76(3.23)	
Yes	56(45.2%)	24.37(3.34)	
Nephrectomy			0.961
No	10(8.1%)	40.79(6.34)	
Yes	114(91.9%)	41.14(2.28)	
Malnutrition-Inflammation Score			
Albumin			0.270
Normal	109(87.9%)	41.77(2.27)	
Low	15(12.1%)	34.06(6.37)	
Hemoglobin			0.001
Normal	89(71.8%)	45.21(2.54)	
Low	35(28.2%)	29.70(3.39)	
Neutrophil: lymphocyte ratio			0.151
>3	47(38.8%)	36.98(3.42)	
≤3	74(61.2%)	43.41(2.83)	
Combined(Alb+Hb+NLR)			0.010*
0 risk	56(45.2%)	47.68(3.20)	
1 risk	47(38.2%)	36.48(3.24)	
≥2 risks	21(16.9%)	32.54(5.07)	

Abbreviations: VATI, visceral adipose tissue index; S.E, standard error; KPS, Karnofsky performance status; Alb, albumin; Hb, hemoglobin; NLR, neutrophil:lymphocyte ratio.

* One-way analysis of variance

## Discussion

This is the first study to use various quantitative (area) and qualitative (density) measurements besides BMI to examine associations between body composition and outcomes of patients with advanced RCC after targeted therapy. Our study indicates that high visceral adiposity correlates strongly with decreased mortality: this was still true after controlling for the Heng criteria and other effect modifiers. Visceral adiposity elucidated a relationship that was not significant when evaluated by BMI; thus, our findings further highlight that body composition measurements are superior to BMI for prognostication.

Growing evidence suggests that BMI is only a rough initial measurement and that including body composition yields more information on an individual level. Amounts of skeletal muscle and adipose tissue have been found to vary widely within each BMI stratum.[21] For instance, both male (61%) and female (107%) patients with metastatic RCC have high coefficients of variation for VAT.[11] Furthermore, body composition has been found to be associated with pathophysiologic phenomena other than cancer. In patients with localized rectal cancer, visceral obesity, but not BMI, is reportedly strongly associated with preoperative metabolic comorbidities.[22]

Few studies have evaluated visceral adiposity and outcomes in patients with advanced RCC in the target therapy era.[21] Ladoire et al. first reported that increased visceral adiposity was associated with increased risk of death (HR: 6.26, 95% CI: 2.29–17.08) in 64 patients with advanced RCC.[9] However, Steffens et al. evaluated 116 such subjects and reported superior outcomes for patients with visceral obesity (HR: 2.97, 95% CI: 1.36–6.47).[10] Recently, Antoun et al. found no significant associations between visceral adiposity and RCC survival.[11] These conflicting results clearly indicate that further study of this issue is required. In the current study, we attempted to better define the prognostic value of visceral adiposity in several ways. First, we used the prognostic factors of the Heng criteria in our multivariate analysis. In contrast, both Ladoire et al. and Steffen et al. used the Memorial Sloan-Kettering Cancer Center, which were developed in the immunotherapy era. Second, we treated the tested predictors as continuous in Cox regression models, and used X-tile software to define the optimal cutoffs for male and female patients. All three studies used median splits to dichotomize measurements. Since this categorization is arbitrary, high and low categories may have different meanings in different samples. Furthermore, dichotomization inevitably results in loss of information and power.[23] Third, we enrolled only patients treated with targeted therapies. Although Antoun et al. studied the largest number of patients (149), nearly 1/3 of their patients received placebos. In previous studies of RCC and colon cancer, visceral adiposity has specifically been associated with outcome in the context of antiangiogenesis therapies.[10, 24] Fourth, unlike the previous studies, we performed thorough multivariate analyses to assess many effect modifiers. The results of our study can be interpreted to mean low VAT is associated with double the risk of death. This finding is consistent with a Japanese study of localized RCC: increasing VAT other than BMI was an independent prognostic factor of better survival.[25]

The mechanism by which visceral obesity may improve survival of patients with advanced RCC is not well understood. Our exploratory analyses suggest that baseline visceral adiposity represents nutritional status. Body-fat depletion is a hallmark of cancer cachexia.[26] Malnutrition is reportedly associated with poorer OS in various malignancies, [27, 28] as well as reduced benefit from medical treatments, [29] poorer tumor response to chemotherapy, [28, 29] and increased chemotherapy-related toxicity.[30] A study using CT scanning showed that cachectic cancer patients with gastrointestinal carcinoma have significantly smaller areas of visceral adipose tissue than control subjects.[31] In a follow-up study of progressive cancer-related cachexia, body composition analysis has shown that body fat is lost more rapidly than lean mass and occurs preferentially from the trunk, followed by leg and arm adipose tissue.[32] Therefore, measurement of visceral adipose tissue may be a more sensitive and accurate estimation of nutritional status than BMI. Furthermore, tumor-derived factors may influence host nutritional status.[30] [33] Tumor-derived tumor necrosis factor-alpha (TNF-α) promotes progression and epithelial-mesenchymal transition in RCC cell lines.[34] Because TNF-α is a key regulator of cancer cachexia, we postulate that undernutrition may be associated with high concentrations of this tumor-derived cytokine in peripheral blood, which also indicates highly aggressive tumors. Taken together, accurate determination of visceral obesity may play a vital role by providing a comprehensive indicator of nutritional status and biological factors that potentially influence outcomes in patients with RCC.

Given the rigorous approach we used and combining our findings with those of previously reported European studies, [9, 10] we suggest there is a positive correlation between VAT area and survival. Measuring VAT area by CT has several advantages: it is non-invasive, highly reproducible, routinely used for cancer surveillance and quick (no more than 5 minutes per patient). Further population-based validation studies are needed to confirm our findings.

The major limitations of our study, as well as of previous studies, are the retrospective design, number of patients, heterogeneous patient population, and relatively short follow-up. We found an association between VAT index and OS only; however, OS is regarded as the most important endpoint for cancer patients. Because most patients in our cohort were classified as low and intermediate risk, our findings should be further tested in high-risk subjects. Moreover, Heng stratification was originally developed only for first-line VEGF therapy and our patients were significantly younger than their typical western counterparts. The mechanisms underlying the favorable prognostic role of large-volume VAT remain speculative. Further analysis of nutritional and endocrine factors, tumor-host interaction, and temporal monitoring of adipose tissue may uncover biological mechanisms for this association and possibly lead to the development of new interventions.

In conclusion, we found that visceral adipose as measured by CT is an independent prognostic factor in Asian patients treated with targeted therapy for advanced RCC. Accurate determination of visceral obesity may play a vital role by providing a comprehensive indicator of nutritional status and biological factors that potentially influence outcomes in patients with RCC.

## Supporting Information

S1 Table Multivariate Cox regression models analysis and final model. (DOCX)
