# Supplementary material for: Prognostic Value of Components of Body Composition in Patients Treated with Targeted Therapy for Advanced Renal Cell Carcinoma: A Retrospective Case Series
Source: PLoS One. 2015 Feb 10;10(2):e0118022. doi: 10.1371/journal.pone.0118022 (PMC4323238; doi:10.1371/journal.pone.0118022)
Supplement: S1 Table — (DOCX) [file pone.0118022.s001.docx]

| **Table S1**. **Multivariate Cox regression models analysis and final model** | | | |
| --- | --- | --- | --- |
| **Parameter** | **Hazard Ratio** | **95% CI** | ***P*-value** |
| KPS < 80% | 2.602 | 1.178-5.747 | 0.018 |
| Hemoglobin < LLN | 2.334 | 1.348-4.259 | 0.003 |
| Calcium > ULN | 2.789 | 1.183-6.578 | 0.019 |
| Low VAT index* | 2.396 | 1.349-4.038 | 0.002 |
| Abbreviations: VAT, visceral adipose tissue; KPS, Karnofsky performance status; LLN, lower limits of normal; ULN, upper limits of normal  *VAT index less than 33.3 cm^2^/m^2^ for male or 17.7 cm^2^/m^2^ for female | | | |
